# Unique Pattern of Enzootic Primate Viruses in Gibraltar Macaques

**DOI:** 10.3201/eid1407.071643

**Published:** 2008-07

**Authors:** Gregory A. Engel, Mark Pizarro, Eric Shaw, John Cortes, Agustin Fuentes, Peter Barry, Nicholas Lerche, Richard Grant, Douglas Cohn, Lisa Jones-Engel

**Affiliations:** * University of Washington National Primate Research Center, Seattle, Washington, USA; † Gibraltar Veterinary Clinic, Gibraltar; ‡ Gibraltar Ornithological and Natural History Society, Gibraltar; § University of Notre Dame, Notre Dame, Indiana, USA; ¶ University of California, Davis, California, USA; # Albany Medical College, Albany, New York, USA; 1 Current affiliation: SNBL USA Ltd., Everett, Washington, USA

**Keywords:** primate zoonoses, simian foamy virus, herpesvirus B, simian T-cell lymphotropic virus, simian retrovirus, rhesus cytomegalovirus, macaques, Gibraltar, Barbary ape, dispatch

## Abstract

Because Gibraltar's macaques (*Macaca sylvanus*) have frequent contact with humans, we assayed 79 macaques for antibodies to enzootic primate viruses. All macaques were seronegative for herpesvirus B, simian T-cell lymphotropic virus, simian retrovirus, simian immunodeficiency virus, and rhesus cytomegalovirus. Seroprevalence of simian foamy virus reached 88% among adult animals.

Animal reservoirs are the most likely sources of emerging infectious diseases that threaten human populations (*1*). Infectious agents from nonhuman primates, in particular, have caused a disproportionate number of new human diseases, leading to calls for international surveillance to monitor the human–nonhuman primate interface. This recognition in turn prompts a need for data focusing on areas and contexts in which humans and nonhuman primates come into contact, creating the potential for zoonotic transmission (*2*,*3*).

Over the past decades, several viruses enzootic to nonhuman primates have been described, and a substantial amount of literature is devoted to their biology, genetics, and capacity to cause disease in humans. The most well known among these, in terms of public health, is simian immunodeficiency virus (SIV), the progenitor of HIV. Other nonhuman primate–borne viruses with known or suspected links to human disease are simian T-cell lymphotropic virus (STLV) and herpesvirus B (Cercopithecine herpesvirus 1) (CeHV-1).

Simian foamy virus (SFV) has received increasing attention as recent findings suggest that human contact with Asian macaques in a number of contexts leads to infection with this virus (*3*,*4*). All free-ranging macaque populations studied to date harbor SFV. Limited data on SFV in humans suggest that infection persists over time. In a small study of 14 humans infected with SFV at primate research centers in the United States, 1 of 7 persons followed consistently over time was shown to have asymptomatic, nonprogressive, monoclonal natural killer cell lymphocytosis (*5*); however, no data link SFV infection with human disease. Further research is needed to determine whether SFV causes immunologic or hematologic effects in humans.

Europe’s only population of free-ranging nonhuman primates is found in the Upper Rock Nature Reserve in Gibraltar. These monkeys, also known as the Barbary ape, are macaques of the species *Macaca sylvanus*. When Gibraltar’s macaque population dwindled dangerously during World War II, the British government introduced *M. sylvanus* from Morocco and possibly Algeria to prevent local extinction. Over the past few decades, protection and provisioning have led to a steady rise in the population, from ≈30–50 to ≈240 currently.

In addition to their status as a unique wildlife heritage, Gibraltar’s macaques are a boon for the local economy. On a typical day, taxi drivers and tour guides ferry thousands of visitors to the Upper Rock Reserve and often use food to entice monkeys to sit on the head or shoulders of visitors (Figure). Each year, >700,000 persons visit the reserve. In recent years, burgeoning macaque populations have become a nuisance for some Gibraltarians living on the edges of the nature reserve, leading to selected culling.

**Figure:**
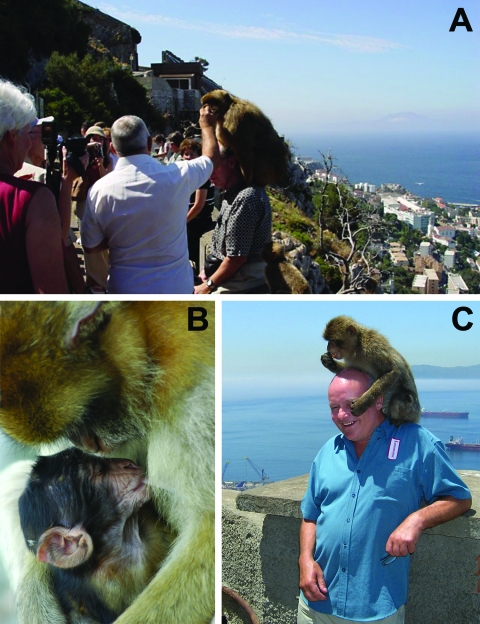
A) Each year >700,000 tourists visit Gibraltar's Upper Rock Reserve, contributing millions of dollars to the local economy. B) Tourists find Gibraltar' macaques compelling. C) Tour guides use food to entice macaques to perch on visitors, potentially exposing the visitors’ mucous membranes to macaque body fluids, a potential route for cross-species transmission of enzootic macaque viruses.

Recent surveys of the Gibraltar macaques have described 6 groups ranging in size from 14 to 64 individuals, with some overlap in range (*6*). Intensity and pattern of contact with humans vary by group; only 3 groups enter into areas visited regularly by tourists. In spite of extensive contact, aggressive interactions with humans are observed less frequently (on average, 170 persons/year are treated for monkey-related injuries) in Gibraltar than in comparable contexts in Asia (*7*). Close human–macaque contact has the potential to transmit infectious agents between the species (*7*,*8*). Given the large amount of interspecies contact occurring in Gibraltar, we sought to characterize the macaques’ potential as a reservoir for zoonotic disease.

## The Study

We began an ongoing longitudinal serosurvey in 2004, sampling macaques from each of Gibraltar’s 6 groups for 6 known enzootic nonhuman primate–borne viruses: SIV, CeHV-1, rhesus cytomegalovirus (RhCMV), STLV, SFV, and simian retrovirus (SRV). Trapping, sampling, and assay protocols have been reported elsewhere (*2*,*9*–*11*).

As of 2007, a total of 79 animals were sampled (Table). None of the animals showed evidence of having been infected with SIV, SRV, an alphaherpesvirus antigenically related to CeHV-1, STLV, or with a CMV antigenically related to RhCMV. The complete absence of seroreactivity to CeHV-1 and RhCMV by ELISA was particularly striking because both viruses are considered enzootic in populations of wild-caught and captive macaques (*9*,*13*). In contrast, more than half of the population showed evidence of infection with SFV (Table). Prevalence of SFV infection increased with age, from 20% among young juveniles to ≈90% among adults. No differences in SFV antibody prevalence were detected between male and female macaques or among the 6 groups. Eight macaques were sampled at 2 periods during the study. None of the 5 initially SFV-seronegative macaques had seroconverted by the second sampling. Similarly, all 3 of the initially SFV-seropositive macaques remained seropositive at second sampling.

**Table:** Seroprevalence of enzootic simian viruses in macaques, Gibraltar, 2004–2007

Category	No. (%)	Seroprevalence, % (no. positive/no. tested)					
		SFV*	SIV†	SRV†	CeHV-1†	STLV†	RhCMV‡
Sex							
Male	45 (57.0)	57.8 (26/45)	0.0	0.0	0.0	0.0	0.0
Female	34 (43.0)	52.9 (18/34)	0.0	0.0	0.0	0.0	0.0
Age class§							
Young juvenile	10 (12.7)	20.0 (2/10)	0.0	0.0	0.0	0.0	0.0
Older juvenile	31 (39.2)	38.7 (12/31)	0.0	0.0	0.0	0.0	0.0
Subadult	13 (16.5)	61.5 (8/13)	0.0	0.0	0.0	0.0	0.0
Adult	25 (31.6)	88.0 (22/25)	0.0	0.0	0.0	0.0	0.0
Total	79	55.7 (44/79)	0.0	0.0	0.0	0.0	0.0

*Simian foamy virus (SFV) seroprevalence for 2004 samples determined using enzyme immunoassay. Subsequent samples screened as part of the whole-virus multiplex flow cytometric assay (*11*). †Simian immunodeficiency virus (SIV), simian retrovirus (SRV), Cercopithecine herpesvirus 1 (CeHV-1), simian T-cell lymphotropic virus (STLV) seroprevalence for 2004 samples determined by using multiplex microbead assay (*10*); seroprevalence for subsequent samples determined by using whole-virus multiplex flow cytometric assay (*11*). ‡Rhesus cytomegalovirus (RhCMV) seroprevalence for the 2004 samples (n = 40) determined by ELISA according to previously published protocols (*12*). Subsequent samples not screened. §Age class determinations made on the basis of dental eruption sequence and morphometrics: young juvenile, <1 y of age; older juvenile, 1–3 y; subadult, 3–5 y; adult, >5 y.

## Conclusions

The Gibraltar macaques are serologically unique. The population shows no evidence of infection with either CeHV-1 or RhCMV, in contrast to other macaque populations studied in which animals seroconvert early in life (*14*). Both these viruses are old viruses that are thought to have coevolved with nonhuman primate species over millions of years. One possible explanation for the lack of CeHV-1 and RhCMV in this population is that the *M. sylvanus* populations in North Africa from which they were derived are not infected with these viruses. At present, the prevalence of these 2 viruses among North African macaques is unknown. If North African *M sylvanus* are infected with CeHV-1 and RhCMV, our results could be explained by a founder effect or the loss of genetic variation when a new colony is established by a very small number of individuals from a larger population. Because seroconversion begins early in life, this hypothesis would imply that the progenitors of the present population were translocated from North Africa when they were young, likely <1 year of age, and therefore less likely to be infected with the viruses.

SFV seroprevalence in Gibraltar macaques mirrors that of other studied macaque populations in South and Southeast Asia with the exception that they seroconvert to SFV at a later age. For example, 81% of free-ranging macaques in Thailand are SFV positive by the age of 3 years, compared with only 38.7% of Gibraltar macaques (*15*). This observation could be explained by decreased rates of aggression because bites are thought to be a principal mode of virus transmission. Properties of the virus or physiologic or immunologic characteristics of the macaques could also influence virus transmission.

The relatively low rates of interspecies aggression observed between macaques and humans in Gibraltar and the low seroprevalence of enzootic nonhuman primate viruses detected in this serosurvey suggest that the risk for virus transmission from macaques to humans in Gibraltar is lower than this risk in other locations where humans come into contact with free-ranging macaques. Nevertheless, the abundance of contact between humans and macaques in Gibraltar implies that the possibility of bidirectional cross-species transmission of infectious agents, including SFV, remains and has implications for humans and macaques. This prospect justifies continued vigilance by local park authorities to monitor and regulate contact between humans and macaques at the Upper Rock Reserve. We recommend strengthened efforts to discourage tourists and locals from feeding macaques and strict enforcement of rules enjoining visitors from seeking contact with them. Macaques should be fed exclusively in designated areas by trained park personnel; such feeding should compensate for decreased feeding by the general public. Finally, water supplies should be enhanced at feeding sites. These changes will preserve the public’s ability to observe the macaques while simultaneously reducing the risk for cross-species transmission of infectious agents.
